# Participants reporting greater desire to have children demonstrate weaker preferences for younger adult faces

**DOI:** 10.1371/journal.pone.0336292

**Published:** 2025-12-03

**Authors:** Jingheng Li, Pengting Lee, Benedict C. Jones, Victor K. M. Shiramizu

**Affiliations:** Department of Psychological Sciences & Health, University of Strathclyde, Glasgow, United Kingdom; Northumbria University, UNITED KINGDOM OF GREAT BRITAIN AND NORTHERN IRELAND

## Abstract

The tendency for men to find younger adult women more attractive has been hypothesised to reflect preferences for cues of reproductive potential. To further test this hypothesis, we investigated the possible relationship between heterosexual men and women’s reported desire to have children and their preferences for cues of youth in face images of potential mates. Contrary to our expectation that reported desire to have children would be correlated with stronger preferences for younger adult faces, and that this pattern of results would be particularly pronounced when men judged women’s attractiveness, both men and women who reported greater desire to have children actually demonstrated weaker preferences for the faces of younger potential mates. Follow-up work suggested that this pattern of results was unlikely to have occurred because individuals with older faces were perceived as more likely to be better parents (Study 2) or wealthier (Study 3). While the role of individual differences in desire to have children in preferences for facial cues of age remains somewhat unclear, our results show no evidence for the widely held view that strong reliable sex differences exist in preferences for cues of youth.

## Introduction

People with more attractive faces tend to be preferred as both romantic and social partners and preferred in hiring and voting decisions (for reviews see [1–3]). Consequently, many studies have sought to identify characteristics in faces that influence attractiveness judgments (for reviews see [2]). Many studies have demonstrated that people can assess age somewhat accurately from face photographs, indicating that faces contain valid cues of age (e.g., [4,5]). Moreover, many studies have shown that people generally judge the faces of younger adults to be more attractive than the faces of older adults (e.g., [6,7]).

Because age-related decline in fertility is considerably more pronounced in women than in men, preferences for cues of youth are predicted to be stronger when men assess women’s suitability as mates than when women assess men’s suitability as mates [8–11]. Consistent with this proposal, this sex difference in preferences for youth has been reported in many studies investigating stated preferences for youth (for reviews see [12]). Given both the role that fertility is hypothesised to play in preferences for cues of youth and the sex difference in age-related decline in fertility, individuals, and men in particular, reporting greater desire to have children might be expected to show stronger preferences for cues of youth in potential mates’ faces. However, to date, there have been no studies directly testing for sex differences in the association between reported desire to have children and preferences for facial cues of youth. Although Moore et al. [13] found that women reporting greater desire to have children showed stronger preferences for men with feminine faces (a characteristic known to influence perceptions of youth [14–16]), they did not test for possible links between male participants’ face preferences and reported desire to have children.

In light of the above, in Study 1 we tested for possible associations between men’s and women’s reported desire to have children (assessed using Rholes et al’s [17] Desire to Have Children questionnaire) and preferences for facial cues of youth. Specifically, we tested the prediction that individuals reporting greater desire to have children would show stronger preferences for facial cues of youth and that this link between desire to have children and preferences for facial cues of youth will be stronger when men assess women’s attractiveness than when women assess men’s attractiveness. To aid in our interpretation of Study 1’s results, we then tested for a possible association between facial cues of youth and the extent to which individuals were perceived as likely to be good parents (Study 2) or wealthy (Study 3). We investigated perceptions of parenting ability and wealth in these latter studies because these factors are hypothesised to play an important role in individual differences in human mate preferences [16,18–20].

## Study 1

Study 1 tested for possible associations between men’s and women’s reported desire to have children (assessed using Rholes et al’s [17] Desire to Have Children questionnaire) and preferences for facial cues of youth. Specifically, we tested the prediction that individuals reporting greater desire to have children will show stronger preferences for facial cues of youth and that this link between desire to have children and preferences for facial cues of youth will be stronger when men assess women’s attractiveness than when women assess men’s attractiveness.

### Methods

All procedures used were approved by the Department of Psychological Sciences and Health (University of Strathclyde) Ethics Commitee (approval number: 27.13.11.2023.A, approval granted on 27 November 2023), all work was undertaken in accordance with the Declaration of Helsinki, and all participants provided written informed consent.

One hundred and forty-nine heterosexual men (mean age = 31.11 years, SD = 6.99 years) and 151 heterosexual women (mean age = 29.98 years, SD = 6.28 years) took part in the study. None of the participants had children, all were resident in the UK, and all reported having English as their first language. Participants were recruited via Prolific and the study was carried out using Qualtrics (from 09/08/2024 to 19/08/2024).

Male participants rated the attractiveness of 50 female faces (mean age = 37.5 years, SD = 13.25 years, range = 19–55 years) and female participants rated the attractiveness of 50 male faces (mean age = 35.06 years, SD = 12.46 years, range = 20–54 years) using a 1 (not at all) to 7 (very) scale. Trial order was fully randomised, and the attractiveness-rating task was self-paced. The face images used in this attractiveness-rating task had been randomly selected from images of individuals between 19 and 55 years of age in an open-access face-image database [4]. Images were full colour and individuals depicted in the images were posed front on to the camera, with neutral expressions, and direct gaze. Clothing was standardised across individuals. Inter-rater agreement was high for attractiveness ratings of both male (Cronbach’s alpha = .993) and female (Cronbach’s alpha = .995) faces. The mean attractiveness rating for male faces was 2.54 (SD = 1.42) and the mean attractiveness rating for female faces was 2.77 (SD = 1.49). Previous work [21] has shown that rated age and actual age are very highly correlated in this sample of faces (r = .96).

Immediately after completing the attractiveness-rating task, participants completed Rholes et al’s [17] Desire to Have Children questionnaire. This questionnaire is a 12-item scale on which participants indicate their level of agreement (on a seven-point scale) with statements such as “I have a strong desire to have children” (Cronbach’s alpha = .89). Higher scores indicate greater desire for children. Female participants (M = 4.32, SD = 1.36) scored slightly higher on the Desire to Have Children questionnaire than did male participants (M = 4.14, SD = 1.25), but this difference was not significant (t = 1.22, p = .22).

### Results

All analyses were carried out using R [22], with the packages tidyverse 1.3.1 [23], psych 2.2.5 [24], jtools 2.2.3 [25], lmerTest 3.1−3 [26], kableExtra 1.3.4 [27], interactions 1.2.0 [28], rstatix 0.7.2 [29], and pander 0.6.5 [30]. All data, full outputs, and analysis code are publicly available on the Open Science Framework (https://osf.io/mu4gz/).

Attractiveness ratings were analysed using a linear mixed effects model. The model included participant sex (effect coded so that +0.5 corresponded to female and −0.5 corresponded to male), desire to have children, stimulus age, and participant age as predictors. The model also included the two-way and three-way interactions shown in Table 1, as well as by-subject random intercepts, by-stimuli random intercepts, by-subject random slopes for stimulus age, and by-stimuli random slopes for desire to have children and participant age. All continuous variables were converted to z scores prior to analyses. Full results for this analysis are summarised in Table 1.

**Table 1:** Summary of results of linear mixed effects model analysing attractiveness ratings in Study 1.

	Estimate	SE	t	df	p
(Intercept)	2.655	0.066	40.005	253.315	< 0.001
participant sex	−0.342	0.133	−2.577	254.645	0.011
desire to have children	−0.071	0.046	−1.556	301.983	0.121
stimulus age	−0.752	0.053	−14.230	123.375	0.000
participant age	0.102	0.045	2.275	316.039	0.024
participant sex x desire to have children	0.176	0.088	1.994	300.855	0.047
participant sex x stimulus age	0.061	0.106	0.573	123.439	0.568
desire to have children x stimulus age	0.036	0.018	1.973	301.604	0.049
participant sex x desire to have children x stimulus age	0.027	0.036	0.755	301.620	0.451

**Fig 1:**
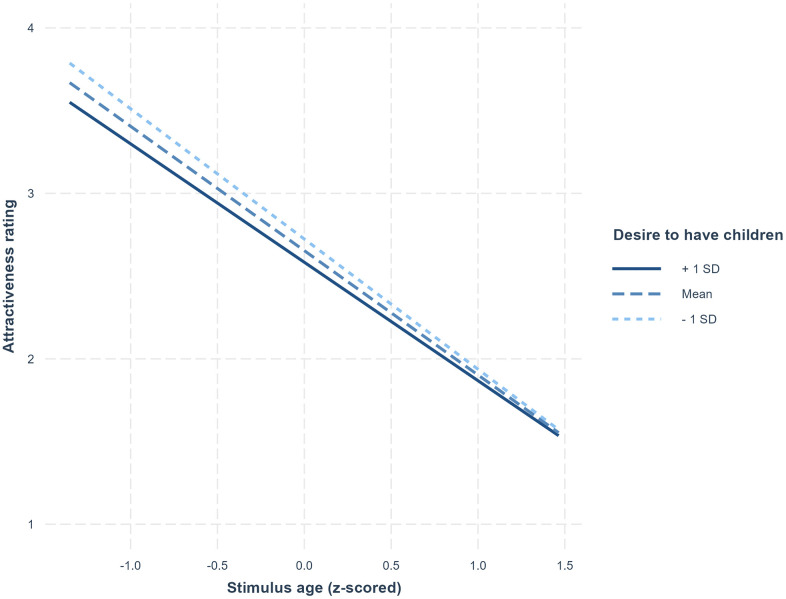
The significant two-way interaction between desire to have children and stimulus age in Study 1.

The main effect of stimulus age was significant, indicating that participants generally rated younger adult faces as more attractive. The significant main effect of participant sex indicated that male participants rated faces as more attractive than did female participants. The significant main effect of participant age indicated that older participants rated faces as more attractive than did younger participants. The two-way interaction between desire to have children and stimulus age was significant (see Fig 1). A simple slopes analysis indicated that the two-way interaction between desire to have children and stimulus age reflected participants who scored higher on desire to have children showing weaker preferences for younger adult faces (see Fig 1). The two-way interaction between participant sex and desire to have children was also significant. Analysing responses separately for male and female participants showed a significant negative effect of desire to have children for male participants and that the effect of desire to have children for female participants was not significant. Full results of analyses undertake to decompose the two-way interactions are given on the OSF (https://osf.io/mu4gz/).

Next, we repeated the analyses described above, this time replacing stimulus age with the mean perceived (i.e., rated) age for each face. Perceived age data for these analyses were taken from Ebner et al’s [4] open-access data. These alternative analyses showed the same pattern of results as our initial analysis (although in this analysis, the interaction between desire to have children and stimulus age was not significant, p = .064, and the interaction between desire to have children and participant sex was not significant, p = .055) and are reported in full at https://osf.io/mu4gz/.

## Study 2

Although we found that participants generally judged younger faces to be more attractive than older faces, we found no evidence that participants who scored higher on the desire to have children questionnaire showed stronger preferences for facial cues of youth. Indeed, our analysis of actual stimulus age found that participants who scored high on the desire to have children questionnaire actually showed significantly weaker preferences for facial cues of youth than did those who scored low on the desire to have children questionnaire. A similar pattern of results was also observed in our analysis of rated (i.e., perceived) stimulus age, although here the interaction between stimulus age and desire to have children was not significant (p = .064). One possible explanation for this potentially surprising pattern of results is that older individuals are perceived to be better parents than younger individuals and that weaker preferences for facial cues of youth among participants reporting greater desire to have children reflects this positive association between age and perceptions of parenting ability. Consistent with this possibility, some previous studies have reported that individuals displaying facial cues of age (i.e., older or older-looking faces) are perceived to be better parents than those displaying facial cues of youth (i.e., younger or younger-looking faces), at least when testing male faces [18]. Consequently, in study 2, we tested for possible relationships between perceptions of parenting ability and stimulus age using the stimuli we used in Study 1.

### Methods

Thirty heterosexual women (mean age = 30.53 years, SD = 6.55 years) and 30 heterosexual men (mean age = 32.67 years, SD = 7.64 years) took part in the study. None of the participants had children, all were resident in the UK, and all reported having English as their first language. Participants were recruited via Prolific and the study was carried out using Qualtrics (from 25/11/2024 to 30/07/2025). Number of raters was based on recommendations in previous work [31,32]. These studies establish that this number of raters per condition is sufficient to produce reliable estimates of face perceptions (i.e., ratings). Note that the larger sample employed in study one’s investigation of individual differences in face preferences is not necessary here because we are not investigating potential individual differences in perceptions.

Male participants rated the 50 female faces from Study 1 and female participants rated the 50 male faces from Study 1 for ‘how likely they are to be a good parent’ using a 1 (very unlikely) to 7 (very likely) scale. Trial order was fully randomised, and the rating task was self-paced. Inter-rater agreement was high for ratings of both male (Cronbach’s alpha = .85) and female (Cronbach’s alpha = .85) faces. The mean rating for male faces was 3.65 (SD = 1.21) and the mean rating for female faces was 3.84 (SD = 1.32).

### Results

All analyses were carried out using R, with the packages tidyverse 1.3.1, psych 2.2.5, jtools 2.2.3, lmerTest 3.1−3, kableExtra 1.3.4, rstatix 0.7.2, and pander 0.6.5 [30]. All data, full outputs, and analysis code are publicly available on the Open Science Framework (https://osf.io/mu4gz/).

Good-parent ratings were analysed using a linear mixed effects model. The model included participant sex (effect coded so that +0.5 corresponded to female and −0.5 corresponded to male), stimulus age, and the interaction between stimulus age and participant sex as predictors. The model also included by-subject random intercepts, by-stimuli random intercepts, and by-subject random slopes for stimulus age. Continuous variables were converted to z scores prior to analyses. Full results for this analysis are summarised in Table 2. No effects were significant, indicating that face age was not significantly correlated with perceptions of being a good parent. Repeating this analysis with rated age in place of actual age showed the same pattern of results (see https://osf.io/mu4gz/ for full results of this alternative analysis).

**Table 2:** Summary of results of linear mixed effects model analysing good-parent ratings in Study 2.

	Estimate	SE	t	df	p
(Intercept)	3.751	0.091	41.394	89.229	< 0.001
participant sex	−0.218	0.181	−1.201	89.229	0.233
stimulus age	−0.120	0.066	−1.824	117.719	0.071
participant sex x stimulus age	0.132	0.132	0.999	117.719	0.320

## Study 3

Results of Study 2 suggested that the tendency for people reporting greater to desire to have children to show weaker preferences for youth (i.e., the pattern of results seen in Study 1) was not due to older individuals being perceived to be better parents. In fact, we found that younger individuals tended to be perceived to be better parents, although this negative effect of age was not significant (p = .071). A different potential explanation of the pattern of results observed in Study 1 is that older individuals are relatively more attractive to people reporting greater desire to have children because older individuals are perceived to be wealthier and, consequently, have more resources available to invest in their family. Consequently, in study 3, we tested for possible relationships between face age and perceptions of wealth.

### Methods

Methods and stimuli were identical to those used in Study 2, except a different group of 30 men (mean age = 32.73 years, SD = 6.67 years) and 30 women (mean age = 32.43 years, SD = 6.22 years) rated the faces for “How wealthy is this person?” on a 1 (not very wealthy) to 7 (very wealthy scale). Participants were recruited to take part in the study from 30/05/2025 to 30/07/2025. Cronbach’s alpha for male faces was.86 (M = 3.73, SD = 1.31) and for female faces was.81 (M = 3.68, SD = 1.42).

### Results

Wealth ratings were analysed in the same was that good-parent ratings were analysed in Study 2. Full results for this analysis are summarised in Table 3. Similar to the findings of Study 2, none of the effects in Study 3 reached statistical significance, indicating that facial age was not significantly correlated with perceptions of wealth. Repeating this analysis with rated age in place of actual age showed a similar pattern of results (see https://osf.io/mu4gz/ for full results of this alternative analysis).

**Table 3:** Summary of results of linear mixed effects model analysing wealth ratings in Study 3.

	Estimate	SE	t	df	p
(Intercept)	3.719	0.079	46.967	112.067	< 0.001
participant sex	0.053	0.158	0.334	112.067	0.739
stimulus age	0.026	0.073	0.350	119.873	0.727
participant sex x stimulus age	0.279	0.147	1.899	119.873	0.060

## Discussion

Consistent with previous research, Study 1’s results suggested that participants generally judged younger adult faces to be more attractive than older adult faces (e.g., [6,7]). However, we found no evidence that this preference for facial cues of youth was stronger when men judged women’s attractiveness than when women judged men’s attractiveness. This null result for the hypothesised sex difference in preferences for facial cues of youth suggests that the sex difference in the importance of youth in mate preferences that has been widely-reported in studies in which participants were asked to rate or rank the importance of various characteristics of potential romantic partners (for reviews see [12]) does not necessarily occur when participants rate the attractiveness of face images. Indeed, recent work examining preferences for youth following blind dates found that men and women showed similar preferences for youthful partners [33].

Many researchers have proposed that preferences for youth reflect preferences for cues of fertility, particularly when men assess the attractiveness of women [8–11]. This claim leads to the prediction that reported desire to have children will be correlated with preferences for facial cues of youth and that this association between reported desire to have children and preferences for facial cues of youth will be stronger when men judge women’s attractiveness than when women judge men’s attractiveness. In Study 1, by contrast with this prediction, our analyses of facial attractiveness judgments found that participants reporting greater desire to have children showed *weaker* preferences for facial cues of youth and that the strength of this association did not differ significantly between when men judged women’s attractiveness and when women judged men’s attractiveness. In other words, regardless of participant sex, participants in our study who reported greater desire to have children showed weaker preferences for facial cues of youth.

Why might people reporting greater desire to have children show weaker preferences for younger adult faces? One possibility is that this pattern of results occurs because older individuals are perceived to be better parents than younger individuals. Indeed, some previous findings support this explanation, finding that adults with older-looking faces were perceived to be better parents (e.g., [18]). However, in Study 2 we found that there was no significant association between face age and perceptions of being a good parent. These results suggest that the effect of desire to have children on preferences for facial cues of youth that we observed in Study 1 is unlikely to reflect older adults being perceived to be better parents. A different possible explanation for the pattern of results seen in Study 1 is that older people are perceived to be wealthier. However, Study 3 revealed no significant relationship between facial age and perceptions of wealth. Thus, Study 3’s results suggest that tendency for both men and women reporting greater desire to have children to show weaker preferences for youth is unlikely to have occurred because older individuals are perceived to be wealthier and have more resources to invest in their family. We suggest that further work is needed to establish whether the positive effect of desire to have children on preferences for older looking faces is robust and, if it is robust, why it occurs.

Our results raise a number of issues that it may be useful to address in future work. First, whether individual differences in desire to have children are relevant to evolutionary theories of mate preferences or are evolutionarily novel remains an open question. On one hand, reliable contraceptives are a relatively recent technological innovation, meaning that individual differences in desire to have children may have little relevance to evolutionary theories of mate preferences because individuals would have had little opportunity to control their number of offspring. On the other hand, there is substantial variation in number of offspring both within and across low-contraception populations, reflecting the influence of ecological conditions, cultural norms, and individual circumstances rather than a uniform “natural” reproductive pattern [34]. Indeed, whether participants already have children may also moderate the effects of desire to have children on preferences for facial cues of age.

Second, the specific cognitions that underpin perceptions of wealth and parenting ability (investigated in Studies 2 and 3) remain unclear. For example, it is not known whether such perceptions reflect impressions of current or potential parenting ability or wealth. This issue is potentially important for understanding the role perceptions of parenting ability and wealth play in mate preferences. Further work is also needed to establish whether reported desire to have children moderates perceptions of parenting ability and wealth.

Third, it is unclear why the results we observed in Study 1 (lack of sex differences in strength of preferences for facial cues of youth and weaker preferences for facial cues of youth among individuals reporting greater desire to have children) differ from those reported in previous work (e.g., [12,13]). Differences between our results and those reported in studies of stated preferences for age [12] may reflect methodological differences (i.e., revealed preferences for age assessed via preferences for facial cues versus explicitly stated preferences for partner age). Moore et al. [13] found that women’s reported desire to have children was correlated with stronger preferences for feminine male faces. This pattern of results may initially seem at odds with our results because feminine faces are generally perceived to be younger than masculine faces (see, e.g., [15]). These apparently contradictory results may reflect methodological differences, however. Moore et al. [13] assessed preferences for femininity in young adult male faces only (faces in their early twenties), whereas we directly assessed preferences for age in adult faces that depicted a much wider range of ages. Similarly, Moore et al. assessed face preferences using manipulated face stimuli and assessed preferences using a forced-choice paradigm, whereas participants in our study rated individual natural (i.e., unmanipulated) face images. This latter point is potentially noteworthy since previous studies suggest these two methods for assessing perceptions of faces can produce very different findings [35–39].

The current work found that individuals reporting greater desire to have children showed weaker preferences for facial cues of youth in potential mates (Study 1). Results of follow-up studies testing for possible associations between age and perceptions of parenting and wealth suggested that the pattern of results observed in Study 1 is unlikely to have occurred simply because older individuals are perceived to be better parents (Study 2) or be wealthier (Study 3). Although the role of individual differences in desire to have children in preferences for facial cues of age remains somewhat unclear, our results, along with other recent work revisiting putative sex differences in preferences for youth, challenge the widely held view that strong reliable sex differences exist in preferences for youth when assessing the attractiveness of potential mates.
